# Are there hardened smokers in low- and middle-income countries? Findings from the Global Adult Tobacco Survey

**DOI:** 10.18332/tid/100631

**Published:** 2019-02-18

**Authors:** Shaoman Yin, Indu B. Ahluwalia, Krishna Palipudi, Lazarous Mbulo, René A. Arrazola

**Affiliations:** 1 Office on Smoking and Health, National Center for Chronic Disease Prevention and Health Promotion, Centers for Disease Control and Prevention (CDC), Atlanta, United States

**Keywords:** tobacco control, MPOWER, hardened smokers, low- and middle-income countries

## Abstract

**INTRODUCTION:**

Hardened smokers are those who do not want to quit, or find it very difficult to quit. This study assessed the prevalence and predictors of hardened smokers in 19 low- and middle-income countries (LMICs).

**METHODS:**

We used nationally representative data from 19 LMICs that conducted the Global Adult Tobacco Survey during 2009–2013. Our analysis is restricted to adults aged ≥25 years. Hardened smokers were defined as daily smokers who smoked for 5 or more years, and who reported the following: no quit attempt in the past year that lasted 24 or more hours; no interest in quitting, or not planning to quit in the next year; and currently smoked within 30 minutes after waking. For each country, the prevalence of hardened smokers was analyzed by sex, age, residence (urban or rural), educational attainment, wealth index, and knowledge of the danger of smoking. Multivariable logistic regression was used to assess predictors of hardened smoking.

**RESULTS:**

Prevalence of hardened smokers among adults (aged ≥25 years) ranged from 1.1% (Panama) to 14.3% (Russia). Among current smokers (aged ≥25 years), the proportion of hardened smokers ranged from 7.5% (Mexico) to 38.4% (Romania). Adjusted odds of hardened smokers were significantly higher for males (9 of 19 countries), smokers aged 65 years or older (12 of 19 countries), adults with lower educational attainment (9 of 19 countries), and no knowledge of the danger of smoking (8 of 19 countries).

**CONCLUSIONS:**

The spectrum of smokers in the LMICs includes hardened smokers and prevalence varies across population groups. Full implementation of proven tobacco control strategies could reduce hardened smoking in LMICs.

## INTRODUCTION

Tobacco use is a major, preventable, public health threat worldwide. The World Health Organization (WHO) estimates that 1 billion people could die of tobacco-related diseases this century if current trends persist^1^. Tobacco use is increasing rapidly among lowand middle-income countries (LMICs). By 2030, more than 80% of the world’s tobacco-related deaths are projected to occur in LMICs^1^.

The leveling-off of smoking prevalence among some high-income countries^2^ suggests that certain tobacco smokers may be more resistant to quitting^3^. For example, the *hardening hypothesis* has been proposed, which postulates that the population of smokers as a whole is becoming more resistant to quitting over time because of unwillingness or inability to quit, or both^4-6^.

Although there is some clinical evidence in support of the hardening hypothesis^7^, several population-based studies suggest that the population of smokers is not hardening^8-12^. Specifically, for each 1% drop in smoking prevalence, quit attempts increased by 0.55% in the United States and remained stable in Europe, which contradicts the hardening hypothesis^10^.

A significant challenge to testing the hardening hypothesis comes from a lack of consensus as to the definition of hardened smokers^13,14^. Hardened smokers have been generally referred to as smokers who are either unable or unwilling to abstain from smoking^6^. The definitions of hardened smokers used in the literature include a mixture of motivational, dependence, and behavioral variables, such as: age 25 years or older, daily smoking, long-term smoking history, quit attempts, quit intentions, nicotine dependence, or social disapproval of smoking^6,13,14^. Costa et al.^13^ assessed six existing definitions of ‘hardcore’ smokers, and found that the estimated prevalence of ‘hardcore’ smokers in Ontario varied considerably from 0.03% to 13.77% with different definitions, which underscores the need for consensus on the best definition of ‘hardcore’ smoker^13^.

Several studies have examined ‘hardcore’ smokers among different countries such as United States^15^, Canada^16^, Italy^17^, the United Kingdom^18,19^, Norway^9^, the Netherlands^8^, Poland^20^, India^21^, Bangladesh^21^, Thailand^21^, Hong Kong of China^22^, and a local rural area of China^23^. These ‘hardcore’ smokers are generally more likely to be older, single, male, and those with lower levels of socioeconomic status^15,17-19^. However, due to variations in survey questions available and different definitions used, it is difficult to assess the magnitudes and patterns of ‘hardcore’ smokers across countries. In this respect, the Global Adult Tobacco Survey (GATS), which collects data on adult tobacco use and tracks key tobacco control indicators through consistent and standardized protocols across different participating countries^24^, provides a unique tool to analyze ‘hardcore’ smokers. Because LMICs comprise countries with the growing burdens of tobacco-related deaths worldwide^1^, we need to assess hardened smoking across these countries to best guide tobacco control policies, planning, and practices. Our study assessed the prevalence and predictors of hardened smokers in 19 LMICs using the most recent available GATS data.

## METHODS

### Data sources

Publically available data came from GATS, which is a nationally representative, cross-sectional, household survey of adults aged 15 years or older. We selected 19 LMICs from the available GATS data based on the World Bank’s country income classification^25^ at the time of the survey. The 19 countries and years of survey were: Argentina (2012), Bangladesh (2009), China (2010), Egypt (2009), India (2010), Indonesia (2011), Malaysia (2011), Mexico (2009), Nigeria (2012), Panama (2013), Philippines (2009), Poland (2010), Romania (2011), Russia (2009), Thailand (2011), Turkey (2012), Ukraine (2010), Uruguay (2009), and Vietnam (2010). Note that the survey year varies by GATS countries and we used the most available data at the time of this study. Response rates in these countries ranged from 65.1% in Poland to 97.7% in Russia, with a median value of 91.8%. This analysis was restricted to respondents aged 25 years or older to align with the existing literature^8,26^.

### Measures

**Figure 1:**
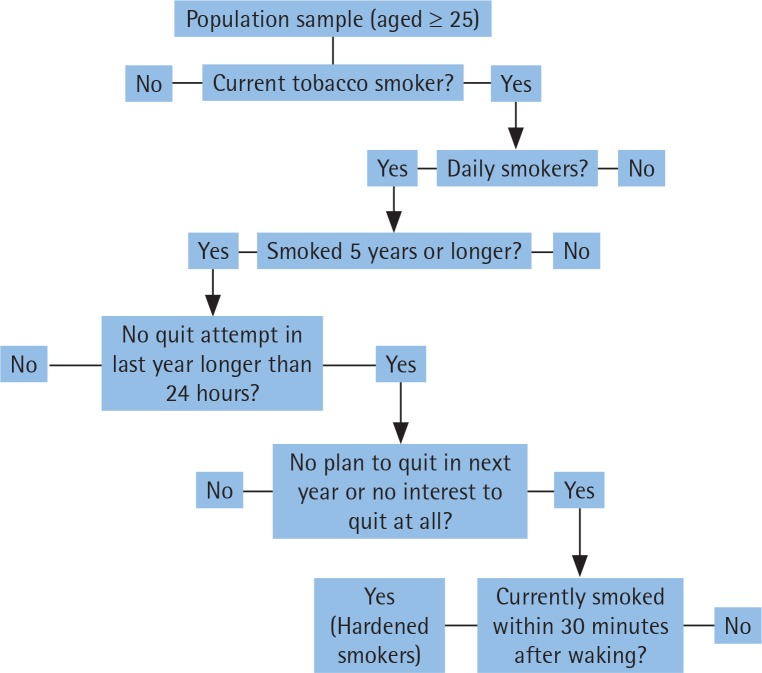
Flowchart for questions to define hardened smokers in 19 LMICs, GATS 2009-2013. Hardened smokers were defined as those who answered yes to all the listed questions.

GATS survey data have been used to identify ‘hardcore’ smokers through two different definitions^20,21^. As there is lack of consensus on defining ‘hardcore’ smokers, we have combined both definitions to develop one that is intended to be more comprehensive as suggested previously^6^. To differentiate our definition from other definitions, we used the term ‘hardened’ smokers instead of ‘hardcore’ smokers. Hardened smokers were defined as those who met the following self-reported criteria: 1) current daily smoking; 2) smoking for 5 or more years; 3) never tried to stop smoking (no quit attempt) in the past 12 months of survey, or last quit attempt lasting 24 or fewer hours; 4) no plan to quit in next 12 months, or not interested in quitting at all; and 5) smoking within 30 minutes after waking up. A flowchart outlining the criteria used to identify hardened smokers is provided in Figure 1. Current smokers were defined as those who currently smoke tobacco daily or less than daily.

Assessed characteristics included in the analyses were: sex (male or female); age (25–44, 45–64, or 65 years or older), residence (urban or rural), educational attainment (low, middle, high), wealth index (low, middle, high), and knowledge of the danger of smoking (yes or no). Due to large variability in educational attainment across countries, the grouping of education levels was performed for each country separately (Supplemental Table S1). Wealth index, a proxy measure for respondent socioeconomic status, was constructed by using principal component analysis using information on household assets^27^. Respondents who answered ‘yes’ to the question of whether smoking tobacco causes serious illness (e.g. lung cancer, heart attack, stroke) were defined as having knowledge of the danger of smoking. Those who answered ‘no’ or ‘don’t know’ were defined as not having knowledge of the danger of smoking.

### Statistical analysis

Data were analyzed by using SAS 9.3-callable SUDAAN to account for the complex survey sampling design of GATS. Among adults aged 25 years or older, three analyses were conducted for each of the 19 LMICs: 1) number and prevalence of hardened smokers, 2) prevalence of current smokers, and 3) proportion and adjusted odds ratio (AOR) of hardened smokers among current smokers. Significance of associations was examined by chi-squared or linear trend tests. Multiple logistic regression analysis was used to determine predictors of hardened smokers, including sex, age, residence, educational level, wealth index, and knowledge on the danger of smoking by country. A p-value of 0.05 or less was considered statistically significant.

## RESULTS

### Numbers and prevalence of hardened smokers

**Figure 2:**
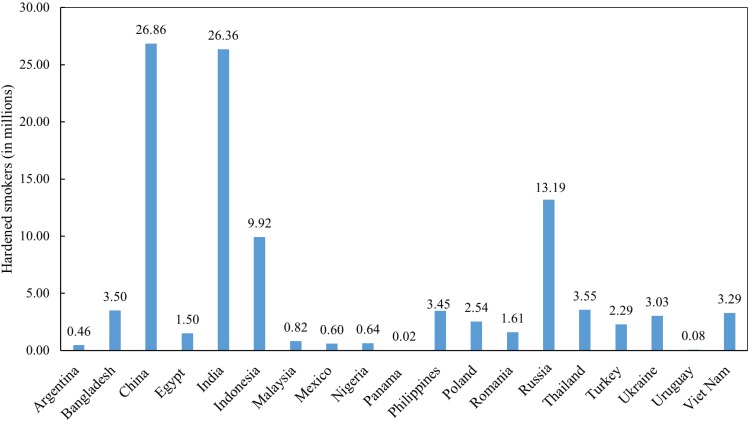
Estimated total number of hardened smokers in millions across 19 LMICs, GATS 2009-2013.

Figure 2 shows the estimated total number of hardened smokers in millions across 19 LMICs, GATS 2009–2013. Overall, the estimated total hardened smokers in the 19 LMICs was 103.7 million. The largest number of hardened smokers were from China (26.9 million), followed by India (26.4 million) and Russia (13.2 million). The least number of hardened smokers were Panama (0.02 million), Uruguay (0.08 million), and Argentina (0.46 million).

**Figure 3:**
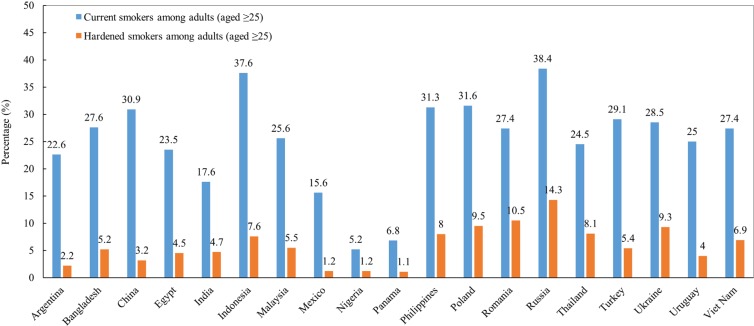
Prevalence of hardened smokers and current smokers among adult population (aged ≥ 25 ) in 19 LMICs, GATS 2009-2013.

Figure 3 describes the prevalence of hardened smokers among adults (aged ≥25 years) across 19 LMICs, GATS 2009–2013. The prevalence of hardened smokers ranged from 1.1% in Panama to 14.3% in Russia. The highest prevalence of hardened smokers was from Russia (14.3%), followed by Romania (10.5%), and Poland (9.5%). The lowest prevalence was found in Panama (1.1%), followed by Mexico (1.2%) and Nigeria (1.2%).

As a comparison, Figure 3 also shows the prevalence of current smokers among adults (aged ≥25 years) across 19 LMICs, GATS 2009–2013. The prevalence of current smokers ranged from 5.2% in Nigeria to 38.4% in Russia. The highest prevalence of current smokers was from Russia (38.4%), followed by Indonesia (37.6%), and Poland (31.6%). The lowest prevalence was found in Nigeria (5.2%), followed by Panama (6.8%) and Mexico (15.6%).

### Characteristics of hardened smokers

We next analyzed the proportions of hardened smokers among current smokers (aged ≥25 years) by each sociodemographic characteristic and knowledge of the danger of smoking for each country (Table 1). Overall, the proportions of hardened smokers among the current smokers ranged from 7.5% in Mexico to 38.4% in Romania (Table 1). In seven of 19 countries, males had greater odds than females to be hardened smokers; no difference was observed in the remaining countries. Distribution of hardened smokers varied by residence, with no specific pattern across the assessed countries. As age increased, the proportion of hardened smokers significantly increased in 12 of 19 countries. In 12 of 19 countries, the proportion of hardened smokers decreased significantly as educational attainment increased. Similarly, in 6 of 19 countries, the proportion of hardened smokers decreased with increasing wealth index. In 6 of 19 countries, the proportion of hardened smokers was significantly higher among those who did not have knowledge of the danger of smoking.

**Table 1:** Proportions of hardened smokers among current smokers (aged ≥25 years) by selected characteristics in 19 LMICs, GATS 2009–2013

*Country (Survey Year) (Sample Size N)*	*Argentina ( 2012 ) (N=1333 )*		*Bangladesh ( 2009 ) (N=2001 )*		*China ( 2010 ) (N=3828 )*		*Egypt ( 2009 ) (N=3713 )*		*India ( 2010 ) (N=10656 )*	
	*% ( 95% CI)*	*p*	*% ( 95% CI)*	*p*	*% ( 95% CI)*	*p*	*% ( 95% CI)*	*p*	*% ( 95% CI)*	*p*
**Overall**	9.9 (6.8–14.3)		19.0 (16.1–22.3)		10.3 (9.2–11.5)		19.1 (17.3–20.9)		26.8 (25.0–28.7)	
**Sex^a^**		0.17		0.21		0.07		0.91		0.21
Male	7.9 (5.1–12.2)		18.6 (15.8–21.8)		10.4 (9.3–11.6)		19.0 (17.3–20.9)		27.5 (25.6–29.5)	
Female	13.5 (7.9–22.0)		30.1 (15.9–49.4)		7.2 (4.6–11.1)		19.8 (9.4–37.1)		21.1 (16.5–26.4)	
**Residence^a^**		NA		0.01		0.24		<0.01		0.31
Urban	NA		13.6 (10.8–16.9)		9.4 (7.7–11.3)		22.2 (19.7–25.0)		25.5 (22.9–28.2)	
Rural	NA		20.7 (17.0–24.9)		11.0 (9.4–12.8)		16.3 (14.1–18.8)		27.2 (25.1–29.5)	
**Age groups^b^**		0.06		0.78		<0.01		0.04		<0.01
25–44	6.2 (3.4–10.9)		17.8 (14.1–22.2)		5.5 (3.7– 8.2)		16.8 (14.1–19.8)		20.6 (17.5–24.1)	
45–64	9.3 (4.1–19.7)		20.4 (15.7–26.0)		10.8 (8.6–13.6)		19.6 (16.9–22.7)		24.5 (21.7–27.5)	
65+	12.7 (7.7–20.3)		18.8 (15.1–23.1)		12.0 (10.3–13.9)		20.4 (18.0–23.0)		30.9 (28.6–33.3)	
**Education^b^**		0.61		<0.01		0.4		0.05		<0.01
Low	7.6 (4.6–12.5)		19.8 (16.7–23.3)		10.2 (8.8–11.8)		20.3 (18.0–22.7)		27.7 (25.7–29.7)	
Middle	17.4 (9.2–30.4)		13.5 (7.9–22.3)		12.2 (9.7–15.2)		17.0 (12.0–23.6)		21.7 (18.1–25.8)	
High	8.1 (4.0–15.6)		3.8 (1.5– 9.4)		6.4 (3.8–10.5)		17.1 (14.7–19.8)		20.3 (15.7–26.0)	
**Wealth index^b^**		0.31		<0.01		0.24		0.84		0.05
Low	13.5 (7.1–24.1)		24.5 (20.1–29.6)		10.0 (7.9–12.5)		19.1 (16.5–21.9)		28.0 (25.4–30.7)	
Middle	7.3 (4.6–11.4)		17.8 (13.9–22.5)		12.6 (10.2–15.4)		19.3 (17.0–21.9)		26.8 (24.3–29.4)	
High	8.6 (5.0–14.3)		10.3 (7.2–14.5)		8.3 (6.4–10.6)		18.4 (15.0–22.3)		23.2 (19.8–26.9)	
**Knowledge of the danger of smoking^a^**		0.70		0.18		0.95		0.24		<0.01
Yes	9.9 (6.7–14.3)		18.7 (15.7–22.0)		10.3 (9.1–11.6)		18.8 (17.1–20.7)		25.3 (23.5–27.2)	
No	12.4 (4.2–31.2)		29.2 (17.1–45.1)		10.4 (7.9–13.6)		25.6 (16.1–38.2)		36.8 (31.9–41.9)

**Table:** 

*Country (Survey Year) (Sample Size N)*	*Indonesia ( 2011 ) (N=2500 )*		*Malaysia ( 2011 ) (N=851 )*		*Mexico ( 2009 ) (N=1407 )*		*Nigeria ( 2012 ) (N=392 )*		*Panama ( 2013 ) (N=857 )*	
	*% ( 95% CI)*	*p*	*% ( 95% CI)*	*p*	*% ( 95% CI)*	*p*	*% ( 95% CI)*	*p*	*% ( 95% CI)*	*p*
**Overall**	20.3 (17.1–24.0)		21.6 (18.0–25.7)		7.5 (5.8– 9.5)		22.5 (16.2–30.4)		16.2 (10.8–23.5)	
**Sex^a^**		0.04		0.03		0.8		NA		0.01
Male	20.7 (17.4–24.4)		22.0 (18.3–26.2)		7.6 (5.9– 9.8)		22.7 (16.2–30.9)		19.4 (12.8–28.4)	
Female	12.4 (6.9–21.3)		7.5 (2.2–23.1)		7.0 (3.8–12.5)		NA		5.7 (2.3–13.3)	
**Residence^a^**		0.56		0.03		0.05		0.02		NA
Urban	19.3 (15.1–24.3)		19.3 (14.8–24.8)		7.9 (6.0–10.3)		12.5 (7.7–19.8)		NA	
Rural	21.3 (16.7–26.7)		27.4 (22.7–32.7)		4.9 (3.1– 7.5)		26.2 (18.0–36.5)		NA	
**Age groups^b^**		0.41		0.72		<0.01		0.89		<0.01
25–44	18.0 (14.0–22.9)		21.5 (15.5–29.1)		4.6 (2.8– 7.4)		21.3 (12.4–34.3)		4.3 (1.8–10.2)	
45–64	23.1 (18.7–28.1)		21.4 (15.4–28.9)		7.8 (4.8–12.4)		26.4 (15.2–41.7)		12.4 (4.9–27.8)	
65+	20.1 (16.8–23.9)		23.2 (17.2–30.5)		10.1 (7.4–13.7)		20.4 (11.8–33.0)		27.1 (18.8–37.5)	
**Education^b^**		<0.01		0.04		0.95		0.65		0.27
Low	23.4 (19.3–28.1)		25.5 (19.6–32.4)		7.6 (5.8–10.0)		22.7 (14.6–33.5)		18.3 (10.8–29.3)	
Middle	16.7 (13.4–20.7)		19.7 (15.0–25.5)		2.2 (0.5– 9.0)		24.3 (15.2–36.6)		15.2 (7.9–27.3)	
High	13.4 (7.6–22.4)		13.3 (5.8–27.5)		8.4 (4.2–16.1)		12.2 (3.7–33.0)		7.6 (2.1–24.0)	
**Wealth index^b^**		0.15		0.15		0.88		0.35		0.21
Low	22.7 (18.1–28.1)		24.5 (19.1–30.9)		5.7 (3.2–10.0)		20.0 (12.8–29.9)		6.1 (2.7–13.0)	
Middle	19.4 (15.7–23.9)		22.1 (16.3–29.3)		8.8 (6.1–12.6)		19.8 (10.6–33.9)		14.9 (6.6–30.3)	
High	18.3 (14.0–23.5)		18.4 (13.0–25.3)		7.0 (5.1– 9.7)		27.9 (16.4–43.1)		18.2 (10.6–29.5)	
**Knowledge of the danger of smoking^a^**		0.19		0.45		0.61		0.48		0.06
Yes	19.4 (16.1–23.2)		21.0 (17.4–25.1)		7.4 (5.7– 9.5)		21.3 (13.4–32.0)		16.8 (11.3–24.4)	
No	24.0 (17.9–31.3)		26.3 (15.2–41.7)		11.1 (2.9–34.4)		26.0 (18.0–36.0)		5.8 (1.5–20.1)

**Table:** 

*Country (Survey Year) (Sample Size N)*	*Philippines ( 2009 ) (N=2315 )*		*Poland ( 2010 ) (N=2184 )*		*Romania ( 2011 ) (N=958 )*		*Russia ( 2009 ) (N=4069 )*		*Thailand ( 2011 ) (N=3873 )*	
	*% ( 95% CI)*	*p*	*% ( 95% CI)*	*p*	*% ( 95% CI)*	*p*	*% ( 95% CI)*	*p*	*% ( 95% CI)*	*p*
**Overall**	25.4 (23.1–27.9)		30.2 (28.0–32.4)		38.4 (34.5–42.6)		37.2 (34.9–39.7)		33.2 (30.6–36.0)	
**Sex^a^**		<0.01		0.08		0.01		<0.01		0.09
Male	28.1 (25.4–30.9)		32.0 (28.9–35.2)		42.0 (37.2–47.0)		42.4 (39.8–45.1)		33.6 (30.9–36.4)	
Female	13.6 (10.1–18.0)		27.7 (24.5–31.1)		31.1 (25.2–37.7)		24.2 (20.5–28.4)		27.9 (21.6–35.2)	
**Residence^a^**		0.16		0.96		0.05		0.02		0.06
Urban	23.4 (19.7–27.6)		30.2 (27.4–33.2)		35.0 (30.1–40.3)		36.1 (33.2–39.1)		30.2 (27.6–32.9)	
Rural	27.0 (24.1–30.0)		30.1 (26.8–33.6)		43.5 (37.3–49.9)		41.0 (38.2–43.9)		34.5 (31.0–38.3)	
**Age groups^b^**		0.04		0.01		0.72		<0.01		<0.01
25–44	22.1 (18.5–26.2)		25.0 (21.2–29.3)		39.0 (30.3–48.5)		29.6 (26.1–33.5)		25.6 (20.8–31.1)	
45–64	26.0 (22.2–30.2)		29.7 (25.4–34.5)		40.2 (32.3–48.6)		35.5 (31.5–39.8)		33.3 (28.9–38.0)	
65+	27.7 (24.0–31.7)		32.7 (29.6–36.0)		37.6 (32.5–43.0)		43.0 (39.6–46.5)		36.9 (33.7–40.1)	
**Education^b^**		0.02		<0.01		0.12		0.05		<0.01
Low	27.1 (24.2–30.1)		33.8 (30.5–37.3)		43.4 (37.5–49.5)		41.8 (38.1–45.7)		36.3 (32.8–39.8)	
Middle	22.6 (18.3–27.6)		28.5 (25.0–32.3)		32.1 (26.2–38.7)		42.8 (39.3–46.4)		28.6 (24.5–33.0)	
High	21.4 (16.7–27.1)		20.5 (15.6–26.5)		37.6 (26.8–49.7)		28.3 (24.8–32.1)		26.6 (21.6–32.2)	
**Wealth index^b^**		0.32		0.03		0.51		<0.01		0.41
Low	25.7 (22.3–29.4)		32.3 (28.4–36.4)		41.7 (34.1–49.7)		44.6 (40.8–48.4)		33.9 (30.1–38.1)	
Middle	27.1 (23.3–31.2)		31.8 (28.2–35.6)		37.3 (30.6–44.4)		37.4 (34.1–40.9)		32.8 (28.9–36.9)	
High	22.2 (18.0–27.0)		26.1 (22.2–30.3)		38.2 (31.9–44.9)		32.0 (28.4–35.7)		31.5 (27.4–35.9)	
**Knowledge of the danger of smoking^a^**		<0.01		<0.01		0.06		0.02		0.98
Yes	23.8 (21.5–26.2)		28.0 (25.7–30.5)		37.4 (33.5–41.6)		36.0 (33.5–38.6)		33.2 (30.7–35.9)	
No	38.6 (30.4–47.6)		39.9 (34.7–45.3)		53.6 (39.9–66.7)		43.5 (37.7–49.6)		33.1 (22.0–46.5)

**Table:** 

*Country (Survey Year) (Sample Size N)*	*Turkey ( 2012 ) (N=2165 )*		*Ukraine ( 2010 ) (N=2106 )*		*Uruguay ( 2009 ) (N=1204 )*		*Vietnam ( 2010 ) (N=2058 )*	
	*% ( 95% CI)*	*p*	*% ( 95% CI)*	*p*	*% ( 95% CI)*	*p*	*% ( 95% CI)*	*p*
**Overall**	18.6 (16.7–20.6)		32.7 (30.2–35.3)		16.1 (13.5–19.1)		25.1 (23.0–27.5)	
**Sex^a^**		0.32		<0.01		0.06		0.36
Male	19.1 (16.9–21.4)		35.1 (32.4–38.0)		18.2 (14.5–22.7)		24.9 (22.7–27.2)	
Female	17.1 (13.9–20.8)		23.2 (18.2–29.1)		13.2 (10.2–17.0)		31.2 (20.0–45.2)	
**Residence^a^**		0.66		0.05		0.73		0.95
Urban	18.8 (16.5–21.2)		31.2 (28.0–34.6)		16.1 (13.3–19.3)		25.0 (22.0–28.3)	
Rural	17.9 (14.9–21.3)		36.2 (32.5–40.1)		16.9 (13.6–20.8)		25.2 (22.4–28.2)	
**Age groups^b^**		<0.01		<0.01		0.59		0.01
25–44	14.1 (11.3–17.5)		24.4 (20.7–28.5)		19.4 (14.1–26.3)		20.6 (16.5–25.3)	
45–64	20.3 (17.0–23.9)		36.1 (31.2–41.4)		9.8 (6.1–15.4)		24.5 (20.8–28.7)	
65+	21.7 (18.4–25.5)		37.0 (33.3–40.8)		16.8 (13.0–21.3)		28.5 (25.0–32.2)	
**Education^b^**		<0.01		<0.01		0.14		<0.01
Low	21.2 (18.5–24.1)		38.9 (34.8–43.2)		17.3 (14.3–20.9)		26.7 (24.2–29.3)	
Middle	19.2 (15.0–24.2)		32.1 (28.5–36.0)		13.7 (8.5–21.5)		20.6 (15.6–26.6)	
High	14.7 (11.9–18.1)		22.1 (17.3–27.7)		12.4 (6.4–22.9)		14.9 (10.4–20.8)	
**Wealth index^b^**		0.14		<0.01		0.45		<0.01
Low	20.8 (17.2–24.8)		38.4 (33.9–43.1)		18.0 (13.6–23.5)		29.4 (25.6–33.6)	
Middle	19.4 (16.5–22.7)		35.3 (31.2–39.7)		16.0 (11.5–21.9)		23.5 (19.8–27.6)	
High	15.6 (12.5–19.4)		24.1 (20.3–28.3)		15.1 (11.1–20.2)		22.4 (18.4–27.0)	
**Knowledge of the danger of smoking^a^**		0.55		<0.01		0.18		<0.01
Yes	18.5 (16.6–20.5)		30.8 (28.3–33.5)		15.6 (13.0–18.5)		23.3 (21.2–25.6)	
No	21.2 (13.7–31.3)		46.4 (39.2–53.6)		25.7 (13.2–43.9)		47.0 (37.9–56.3)	

LMICs: low– and middle–income countries, GATS: Global Adult Tobacco Survey, CI: confidence interval, NA: not available.

a p–values from chi– squared test.

b p–values from linear trend test.

### Predictors of hardened smokers

Among the current smokers, sex, age, residence, educational level, wealth index, and knowledge of the danger of smoking were predictors of hardened smoking, with variations across countries. More specifically, in 9 of 19 LMICs, male smokers had significantly higher odds of hardened smoking than female smokers (Table 2). The magnitude of associations between sex and hardened smoking varied across countries, ranging from 1.4 (95% CI: 1.4–2.0) in Thailand to 5.9 (95% CI: 1.3–27.0) in Malaysia. Residence (urban or rural) was not significantly associated with hardened smoking in most countries except Nigeria (AOR=3.1, 95% CI: 1.3–7.3). Compared to adults aged 25–44 years, those who were older had significantly higher odds of hardened smoking. In 9 of 19 LMICs, odds of hardened smoking were significantly higher for smokers with the lowest education level compared to the highest educational level. Similarly, in 4 of 19 LMICs, hardened smoking was significantly higher among those with lower wealth index. In 8 of 19 LMICs, the odds of hardened smoking were greater among smokers without knowledge of the danger of smoking.

**Table 2:** Predictors of hardened smokers among current smokers (aged ≥25 years) in 19 LMICs, GATS 2009–2013

*Country (Survey Year)*	*Argentina ( 2012 )*	*Bangladesh ( 2009 )*	*China ( 2010 )*	*Egypt ( 2009 )*	*India ( 2010 )*	*Indonesia ( 2011 )*
*AOR ( 95% CI)*						
**Sex**						
Male	0.6 (0.3-1.1)	0.7 (0.3-1.6)	1.6 (1.0-2.6)*	1.1 (0.5-2.6)	1.7 (1.2-2.3)*	1.8 (0.9-3.6)
Female	1.0	1.0	1.0	1.0	1.0	1.0
**Residence**						
Urban	1.0	1.0	1.0	1.0	1.0	1.0
Rural	NA	1.4 (0.9-1.9)	1.3 (0.9-1.7)	0.6 (0.5-0.8)	1.0 (0.8-1.2)	1.0 (0.6-1.5)
**Age groups**						
25–44	1.0	1.0	1.0	1.0	1.0	1.0
45–64	1.7 (0.6-4.8)	1.2 (0.9-1.6)	2.1 (1.3-3.6)*	1.2 (0.9-1.5)	1.2 (1.0-1.5)*	1.3 (1.0-1.8)*
65+	2.3 (1.1-5.0)*	1.1 (0.7-1.5)	2.5 (1.6-3.9)*	1.2 (0.9-1.5)	1.7 (1.3-2.1)*	1.0 (0.7-1.3)
**Education**						
Low	0.7 (0.3-2.1)	3.3 (1.2-9.5)*	1.1 (0.6-2.1)	1.3 (1.0-1.6)*	1.3 (0.9-1.9)	2.2 (1.1-4.3)*
Middle	2.3 (0.8-6.4)	3.4 (1.0-11.5)*	1.6 (0.9-2.9)	1.0 (0.6-1.6)	1.0 (0.7-1.5)	1.3 (0.7-2.5)
High	1.0	1.0	1.0	1.0	1.0	1.0
**Wealth index**						
Low	2.1 (0.8-5.6)	2.4 (1.5-3.8)*	1.2 (0.7-2.0)	1.1 (0.8-1.5)	1.2 (0.9-1.5)	1.0 (0.7-1.5)
Middle	0.8 (0.3-1.8)	1.6 (1.0-2.7)*	1.6 (1.1-2.4)*	1.1 (0.8-1.4)	1.1 (0.9-1.5)	0.9 (0.6-1.3)
High	1.0	1.0	1.0	1.0	1.0	1.0
**Knowledge of the danger of smoking**						
Yes	1.0	1.0	1.0	1.0	1.0	1.0
No	1.6 (0.4-6.1)	1.8 (0.9-3.8)	0.9 (0.6-1.4)	1.5 (0.8-2.7)	1.6 (1.3-2.1)*	1.1 (0.7-1.7)

**Table:** 

*Country (Survey Year)*	*Malaysia ( 2011 )*	*Mexico ( 2009 )*	*Nigeria ( 2012 )*	*Panama ( 2013 )*	*Philippines ( 2009 )*	*Poland ( 2010 )*
*AOR ( 95% CI)*						
**Sex**						
Male	5.9 (1.3-27.0)*	1.1 (0.6-2.2)	1.7 (0.3-9.8)	5.5 (1.6-18.5)*	2.8 (1.9-4.1)*	1.1 (0.9-1.4)
Female	1.0	1.0	1.0	1.0	1.0	1.0
**Residence**						
Urban	1.0	1.0	1.0	1.0	1.0	1.0
Rural	1.4 (0.9-2.1)	0.6 (0.3- 1.1)	3.1 (1.3-7.3)*	NA	1.1 (0.8-1.5)	0.9 (0.7-1.1)
**Age groups**						
25–44	1.0	1.0	1.0	1.0	1.0	1.0
45–64	0.9 (0.5-1.6)	1.7 (0.9-3.3)	1.4 (0.6-3.2)	2.7 (0.7-10.6)	1.3 (0.9-1.7)	1.2 (0.9-1.6)
65+	0.8 (0.5-1.5)	2.4 (1.4-4.3)*	0.7 (0.3-1.7)	9.5 (2.8-32.7)*	1.5 (1.1-2.0)*	1.3 (1.0-1.7)*
**Education**						
Low	2.3 (0.8-6.7)	0.8 (0.4-1.7)	2.4 (0.5-12.5)	3.5 (0.5-23.2)	1.2 (0.8- 1.7)	1.8 (1.2-2.7)*
Middle	1.6 (0.6-4.4)	0.2 (0.0-1.2)	2.0 (0.4-9.0)	3.1 (0.5-18.5)	1.0 (0.6-1.5)	1.4 (1.0-2.1)*
High	1.0	1.0	1.0	1.0	1.0	1.0
**Wealth index**						
Low	1.1 (0.6-2.0)	0.9 (0.4-1.9)	0.3 (0.1-1.1)	0.1 (0.0- 0.5)	1.1 (0.7-1.5)	1.1 (0.8-1.5)
Middle	1.0 (0.6-1.7)	1.3 (0.8-2.0)	0.4 (0.2-1.0)	0.4 (0.1-1.5)	1.2 (0.9-1.6)	1.2 (0.9-1.6)
High	1.0	1.0	1.0	1.0	1.0	1.0
**Knowledge of the danger of smoking**						
Yes	1.0	1.0	1.0	1.0	1.0	1.0
No	1.3 (0.6-2.7)	1.2 (0.3-5.9)	1.6 (0.8-3.2)	0.6 (0.1-3.2)	2.0 (1.3-2.9)*	1.7 (1.3-2.2)*

**Table:** 

*Country (Survey Year)*	*Romania ( 2011 )*	*Russia ( 2009 )*	*Thailand ( 2011 )*	*Turkey ( 2012 )*	*Ukraine ( 2010 )*	*Uruguay ( 2009 )*	*Vietnam ( 2010 )*
*AOR ( 95% CI)*						
**Sex**							
Male	1.5 (1.0-2.2)*	2.2 (1.7-2.8)*	1.4 (1.0-2.0)*	1.2 (0.9-1.6)	1.6 (1.1-2.4)*	1.4 (0.9-2.1)	1.1 (0.6-2.1)
Female	1.0	1.0	1.0	1.0	1.0	1.0	1.0
**Residence**							
Urban	1.0	1.0	1.0	1.0	1.0	1.0	1.0
Rural	1.3 (0.9-2.1)	0.9 (0.8-1.1)	1.1 (0.9-1.4)	0.8 (0.6-1.1)	0.9 (0.7-1.2)	0.9 (0.6-1.4)	0.8 (0.6-1.1)
**Age groups**							
25–44	1.0	1.0	1.0	1.0	1.0	1.0	1.0
45–64	1.0 (0.6-1.6)	1.3 (1.0-1.7)*	1.3 (1.0-1.8)*	1.6 (1.1-2.2)*	1.6 (1.2-2.3)*	0.5 (0.2-0.9)	1.2 (0.9-1.8)
65+	0.9 (0.6-1.4)	1.6 (1.3-2.0)*	1.5 (1.1-2.1)*	1.7 (1.2-2.4)*	1.4 (1.1-1.9)*	0.8 (0.5-1.3)	1.6 (1.1-2.2)*
**Education**							
Low	1.1 (0.6-2.0)	1.3 (1.0-1.7)*	1.4 (1.0-2.0)*	1.3 (1.0-1.8)*	1.7 (1.2-2.5)*	1.4 (0.7-3.0)	1.9 (1.2-3.0)*
Middle	0.7 (0.4-1.3)	1.6 (1.2-2.0)*	1.1 (0.8-1.6)	1.3 (0.9-1.9)	1.4 (1.0-2.0)*	1.1 (0.4- 3.0)	1.5 (0.8-2.5)
High	1.0	1.0	1.0	1.0	1.0	1.0	1.0
**Wealth index**							
Low	0.8 (0.5-1.3)	1.5 (1.2-2.0)*	0.9 (0.7-1.3)	1.4 (1.0-2.0)*	1.5 (1.1-2.2)*	1.1 (0.6-1.8)	1.3 (0.9-1.9)
Middle	0.8 (0.5-1.2)	1.3 (1.0-1.6)*	0.9 (0.7-1.2)	1.3 (1.0-1.8)*	1.5 (1.1-2.0)*	1.0 (0.6-1.6)	1.0 (0.7-1.5)
High	1.0	1.0	1.0	1.0	1.0	1.0	1.0
**Knowledge of the danger of smoking**							
Yes	1.0	1.0	1.0	1.0	1.0	1.0	1.0
No	1.9 (1.0-3.4)*	1.4 (1.1-1.8)*	1.0 (0.6-1.7)	1.1 (0.7-2.0)	1.7 (1.2-2.3)*	2.2 (1.0-4.7)*	2.9 (2.0-4.3)*

LMICs: low- and middle-income countries, GATS: Global Adult Tobacco Survey, CI: confidence interval, NA: not available. Adjusted odds ratio (AOR) was adjusted with all listed characteristics using multiple logistic regression. The dependent variable was hardened smokers (‘yes’ for hardened smokers, ‘no’ for all the rest as non-hardened smokers).

* p≤0.05.

## DISCUSSION

The findings from this study indicate that there are hardened smokers in LMICs and that the prevalence of hardened smoking varies across countries. For example, in 3 of the 19 assessed countries, the overall prevalence of those classified as hardened smokers was relatively low: less than 2.0%. However, 4 of 19 LMICs had prevalence exceeding 9.0%. These findings underscore the importance of fully implementing proven tobacco control strategies that reach all tobacco smokers, particularly hardened smokers.

Studies from high-income countries suggest that the proportion of hardened smokers has not changed considerably, or has even declined in some instances^8-12^. Our study found high proportions (over 20%) of hardened smoking among current smokers in 11 of 19 LMICs. In addition, large numbers of hardened smokers (over 13 million) were observed in some countries, including China, India, and Russia. However, patterns of hardened smoking in LMICs remain uncertain. Continual monitoring of tobacco use patterns in LMICs, particularly the trajectories of hardened smoking, is critical to guide tobacco control policies, planning, and practices. When implemented, GATS provides LMICs with the opportunity to monitor and track the changes and trajectories of hardened smoking, especially when GATS is used regularly.

Consistent with previous studies^15,17-19^, we found that being male, of older age, with lower education, and lower economic status were significant predictors of hardened smoking, with variations across countries. For example, most of the selected characteristics (except residence) were significant predictors for hardened smoking in Russia and Ukraine, whereas only age was found to be significant in predicating hardened smoking in Argentina, Mexico, and Panama. These findings suggest that interventions to address tobacco use among those in old age, males, with lower education, and lower economic status, or mainly the low socioeconomic status (SES) population, are critical. Given the high proportion of hardened smoking among the low SES population, efforts to reduce smoking in this population could help reduce overall rates of tobacco smoking in these countries.

The evidence-based interventions outlined in the WHO Framework Convention on Tobacco Control (FCTC) MPOWER package could be implemented in a way to reach hardened smokers^28^. For instance, health care providers or systems could enhance access to cessation services to ensure that they reach all smokers in their populations, including cessation counseling from a health professional and pharmacotherapy. With appropriate tobacco control programs/policies, even hardened smokers with psychological distress can successfully quit smoking and reduce consumption over time^12^. It is important to tailor these services to country-specific conditions and cultures. Coupling cessation strategies with other population-based tobacco control interventions, as outlined in the WHO’s MPOWER package, could also reduce the prevalence of hardened smoking in LMICs. Increased tobacco product prices are effective at reducing smoking rates among individuals with lower socioeconomic status, and could reduce smoking among hardened smokers^29^.

Knowledge of the danger of smoking is an independent predictor of hardened smoking. Our study found that there are high proportions of hardened smokers among adults without knowledge of the danger of smoking, including those from Russia, Romania, and Poland. This lack of knowledge highlights an opportunity for increasing awareness of the danger of smoking, which has been proven as an effective tobacco control strategy^30^. Article 11 of FCTC, a global health treaty ratified by over 181 countries, requires countries to adopt and implement large, clear, and rotating health warning labels on all tobacco products^28^. However, a recent study shows that the extent of compliance with key requirements for health warning labels varied significantly across LMICs^31^. Implementing and complying with Article 11 of FCTC would be expected to help increase awareness and knowledge of the danger of smoking.

### Limitations

The findings of this study are subject to some limitations. First, the analysis categorizes smokers as not hardened smokers if a respondent refused or had missing information for at least one of the five questions used to define hardened smoking, which may lead to an underestimation of our prevalence estimates. Second, the self-reported data are subject to recall and social desirability biases. The measurements of hardened smokers are not verified through biochemical markers and thus may be subject to uncertainty. Third, due to the limited survey questions in GATS, we may miss some potential predictors for hardened smokers, such as geographical location, country culture, and the implementation level of anti-smoking regulations in the country. Fourth, the survey year varies across countries as GATS is conducted by each country separately though using the same protocol and questions. However, considering the unwillingness of hardened smokers in quitting smoking, their prevalence may be relatively stable in the 4-year study period from 2009 to 2013. Studies using the newly available GATS data to track hardened smokers are warranted. Finally, the questionnaire’s content limited the extent to which certain sociodemographic variables could be assessed. For example, the wealth index is based on the household possessions available in the country data, and these items may not be truly representative of wealth across all countries.

## CONCLUSIONS

This study provides data on the magnitude and patterns of hardened smoking in 19 LMICs. Understanding and reducing hardened smoking is an important factor in reducing tobacco use globally, particularly in LMICs. Fully implementing the evidence-based strategies outlined in the MPOWER package could reduce hardened smoking in LMICs.
