# Supplementary material for: Are there hardened smokers in low- and middle-income countries? Findings from the Global Adult Tobacco Survey
Source: Tob Induc Dis. 2019 Feb 18;17:11. doi: 10.18332/tid/100631 (PMC6751971; doi:10.18332/tid/100631)
Supplement: Supplementary file 1 [file TID-17-11-s1.pdf]

Supplementary Table. Levels of Educational Attainments in 19 LMICs , GATS 2009–2013

Country (survey year)	Low	Middle	High
**Nigeria (2012)**			
	No formal schooling	Junior secondary school completed	College/university completed
	Less than primary school completed	Senior secondary school completed	
	Complete primary education	Less than college/university completed	
**Argentina (2012)**			
	No formal education	Complete secondary education	Incomplete tertiary or university education
	Incomplete primary education		Complete tertiary or university education
	Complete primary education		Special education
	Incomplete secondary education		
**Panama (2013)**			
	No formal schooling	Secondary school completed	College/university completed
	Special education	Vocational	Post-graduate degree completed
	Less than primary school completed	Superior no university	
	Primary school completed		
	Less than secondary school completed		
**Mexico (2009)**			
	Without formal schooling	Technical high school	Normal upper-level
	Primary	Technical or trade degree	Undergraduate
	Secondary		Master/Doctorate
	Technical or trade secondary		
	Normal basic		
	Preparatory or vocational		
**Uruguay (2009)**			
	No formal schooling	Secondary Bachelaurate (4th to 6th)	Primary, secondary teaching degree
	Standard primary school	Utu technical Bachelaurate (4th to 6th)	University or similar
	Special Primary School	Technical education	Tertiary not university
	Basic cycle of high school or utu 1st to 3rd)	Post-graduate	
**Bangladesh (2009)**			
	No formal schooling	Secondary school completed	College/university completed
	Less than primary school completed	High school completed	Post-graduate degree completed
	Primary school completed		
**India (2010)**			
	No formal schooling	Secondary school completed	College/university completed
	Less than primary school completed	Higher school completed	Post-graduate degree completed
	Primary school completed		
	Less than secondary school		
**Indonesia (2011)**			
	Less than primary school completed	Secondary school completed	College/university completed
	Primary school completed	High school completed	Post-graduate degree completed
**Thailand (2011)**			
	No education/illiterate	Grade 9 completed	Certificate/vocational education
	Less than primary school completed	More than grade 9 but less than grade 12	Bachelor degree
	Primary school completed	Grade 12 completed /vocational education	Higher than Bachelor degree
	More than grade 6 but less than grade 9		

Continued

Supplementary Table. Continued

Country (survey year)	Low	Middle	High
**Poland (2010)**			
	No formal education	Secondary (high school, technical college)	Bachelor
	Incomplete elementary	Junior college	Higher (starting with Masters degree)
	Elementary		
	Junior High School		
	Vocational		
**Romania (2011)**			
	No formal education	High school completed	College completed (short term university)
	Primary school completed (4 grades)	Post high school completed	University
	Secondary school completed (grades 5-8)		Post-graduate degree completed
	Grades 9-10 of high school completed		
**Russian Federation (2009)**			
	No formal education	Vocational school/trade school	Some college
	Primary school		College
	Some high school		Advanced degree
	High school		
**Turkey (2012)**			
	Not graduated	Primary education	High school or equivalent
	Elementary school	Secondary or vocational secondary school	College or faculty
			Master or doctor
**Ukraine (2010)**			
	No formal education	High school completed, incomplete professional & vocational secondary education	College/university completed
	Primary school		Post-graduate degree completed
	Less than secondary school completed (<9 grades)		
	Basic secondary school completed (full 9 grades)		
	Full secondary school completed (11 grades)		
**Egypt (2009)**			
	No formal education	Complete high school/equivalent education	Diploma
	Attended, but not finished primary school		Completed university/college
	Complete primary school		Post-graduate degree obtained
	attended, but not finished preparatory school		
	Complete preparatory school		
	attended, but not finished high school		
**China (2010)**			
	No formal schooling	High school/technical secondary school	College/university completed
	Less than primary school completed		Post-graduate degree completed
	Primary School Completed		
	Less than secondary school completed		
	Secondary school completed		

Continued

Supplementary Table. Continued

Country (survey year)	*Low*	*Middle*	*High*
Malaysia (2011)			
	No formal schooling	Secondary school completed	College/university completed
	Less than primary school completed	High school completed	Post-graduate degree completed
	Primary school completed		
	Less than secondary school completed		
Philippines (2009)			
	No formal schooling	High school graduate	College undergraduate
	Elementary undergraduate	Post-secondary, includes in years 1, 2, or 3	College graduate
	Elementary graduate		Post-graduate degree completed
	High school undergraduate		
Vietnam (2010)			
	No formal education	Having completed secondary school	Graduated University/college/ specialized secondary education
	Not having completed primary education		Having been post-graduated
	Having completed primary education		
	Having completed basic secondary school		

© 2019 Yin S.
